# Neither Excessive Nitric Oxide Accumulation nor Acute Hyperglycemia Affects the *N*-Acetylaspartate Network in Wistar Rat Brain Cells

**DOI:** 10.3390/ijms21228541

**Published:** 2020-11-12

**Authors:** Marlena Zyśk, Piotr Pikul, Robert Kowalski, Krzysztof Lewandowski, Monika Sakowicz-Burkiewicz, Tadeusz Pawełczyk

**Affiliations:** 1 Department of Molecular Medicine, Medical University of Gdansk, 80-211 Gdansk, Poland; monika.sakowicz-burkiewicz@gumed.edu.pl (M.S.-B.); tadeusz.pawelczyk@gumed.edu.pl (T.P.); 2 Laboratory of Molecular and Cellular Nephrology, Polish Academy of Science, 80-308 Gdansk, Poland; piotr.pikul@gumed.edu.pl (P.P.); robert.kowalski@gumed.edu.pl (R.K.); 3 Clinical Laboratory University Clinical Center in Gdansk, 80-211 Gdansk, Poland; krzysztof.lewandowski@gumed.edu.pl

**Keywords:** aspartate *N*-acetyltransferase, streptozotocin, SNAP, primary neurons, neural stem cells, NAA

## Abstract

The *N*-acetylaspartate network begins in neurons with *N*-acetylaspartate production catalyzed by aspartate *N*-acetyltransferase from acetyl-CoA and aspartate. Clinical studies reported a significant depletion in *N*-acetylaspartate brain level in type 1 diabetic patients. The main goal of this study was to establish the impact of either hyperglycemia or oxidative stress on the *N*-acetylaspartate network. For the in vitro part of the study, embryonic rat primary neurons were treated by using a nitric oxide generator for 24 h followed by 6 days of post-treatment culture, while the neural stem cells were cultured in media with 25–75 mM glucose. For the in vivo part, male adult Wistar rats were injected with streptozotocin (65 mg/kg body weight, ip) to induce hyperglycemia (diabetes model) and euthanized 2 or 8 weeks later. Finally, the biochemical profile, NAT8L protein/*Nat8l* mRNA levels and enzymatic activity were analyzed. Ongoing oxidative stress processes significantly affected energy metabolism and cholinergic neurotransmission. However, the applied factors did not affect the *N*-acetylaspartate network. This study shows that reduced *N*-acetylaspartate level in type 1 diabetes is not related to oxidative stress and that does not trigger *N*-acetylaspartate network fragility. To reveal why *N*-acetylaspartate is reduced in this pathology, other processes should be considered.

## 1. Introduction

**Figure 1:**
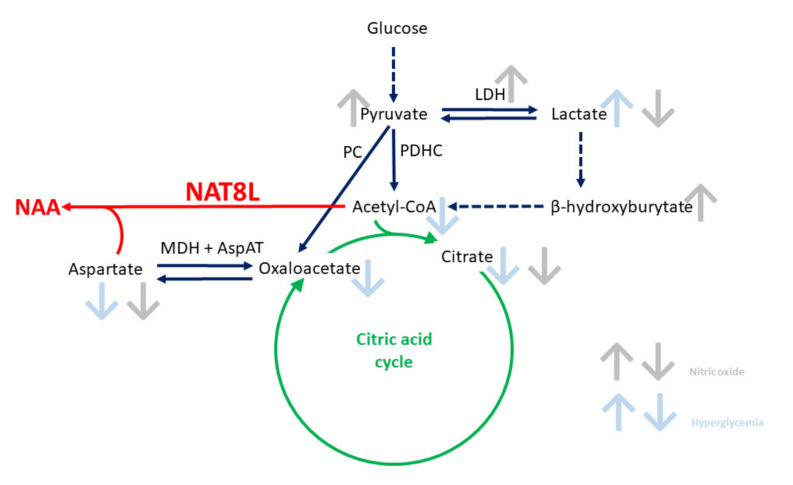
Graphic presentation of the background of this study background: the impact of neurotoxic factors on the *N*-acetylaspartate network related to neuronal energy pathways. Abbreviations: AspAT: aspartate transferase; LDH: lactate dehydrogenase; MDH: malate dehydrogenase; NAA: *N*-acetylaspartate; NAT8L: aspartate *N*-acetyltransferase (or Shati protein); PC: pyruvate carboxylase; PDHC: pyruvate dehydrogenase complex.

*N*-acetylaspartate (NAA) is a brain amino acid mainly produced by neurons from acetyl-CoA and aspartate in the presence of aspartate *N*-acetyltransferase (NAT8L, Shati protein) [1,2,3,4,5,6,7,8,9]. Apart from NAA production, acetyl-CoA is consumed in the mitochondrial tricarboxylic acid cycle, while aspartate has to be shared with the malate–aspartate shuttle (Figure 1) [1,2,3,4,5,6,7,8,9]. *N*-acetylaspartate is used by neurons to conduct crosstalk with glial cells, thus NAA disturbances reflect dysregulations of brain homeostasis [1,2,3,4,5,6,7,8,9,10,11,12,13,14,15]. A couple of diseases listed as involving NAA disturbances share a few molecular pathomechanisms (such as the upregulation of oxidative stress), although they have a wide clinical background, and no connections with NAT8L have been discussed [1,2,3,4,5,6,7,8,9].

Type 1 diabetes mellitus (T1D) is an insulin-dependent chronic metabolic disease that most often develops during childhood and is caused by autoimmune reaction leading to impaired insulin production [16,17,18]. Lack of the endogenous insulin resulted in an abnormal elevation of blood glucose level [16,17,18]. Uncontrolled hyperglycemia might evoke a metabolic crisis, such as lactic acidosis, ketoacidosis, and finally oxidative stress [16]. Moreover, several clinical studies reported significantly lower brain NAA level measured in type 1 diabetic patients [16,17,18]. Since the NAA network interweaves energy pathways that are susceptible to oxidative stress, we assumed that the NAA network will be vulnerable to these conditions as well. Therefore, our overall goal was to identify biochemical variables that might decrease the NAA brain level in the progression of type 1 diabetes. To separate the impact of oxidative stress and hyperglycemia effects on NAA network, the investigated in vitro models were exposed to either a high concentration of nitric oxide from a generator (model of oxidative stress) or to acute hyperglycemia. Streptozotocin-injected Wistar rats were sacrificed as in vivo model of type 1 diabetes. As results, our studies revealed that oxidative stress is not an up- or downregulating factor for the NAA network. We also noted that brain cells can handle shortages of *N*-acetylaspartate substrates. Therefore, we hypothesize that to regulate the NAA network, NAT8L enzymatic activity or gene expression have to be regulated rather than substrate shortages.

## 2. Results and Discussion

### 2.1. Primary Cell Cultures

**Figure 2:**
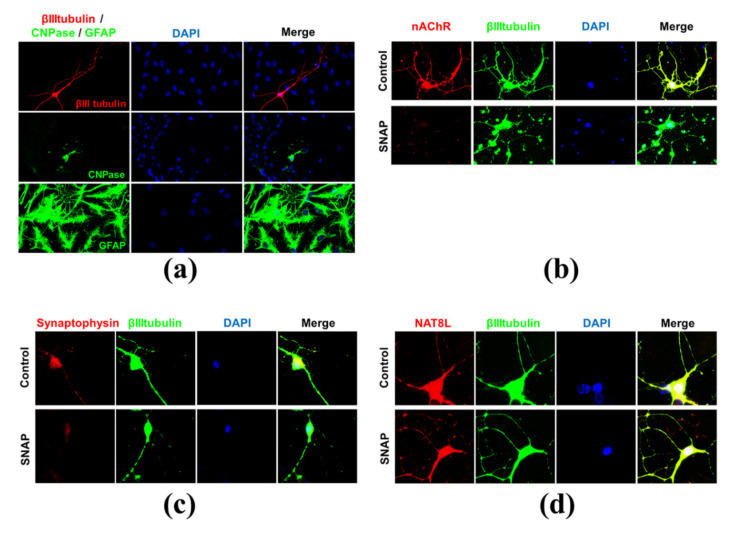
The immunostaining made for the purposes of this project: (**a**) cell types recognized in the neural stem cells model stained by the antibody against β-III-tubulin (neuronal marker), CNPase (2′,3′-Cyclic-nucleotide 3′-phosphodiesterase, oligodendrocytic marker) and glial fibrillary acidic protein (GFAP) (astrocytic marker). The impact of 0.2 mM SNAP (*S*-Nitroso-*N*-acetylpenicillamine) on primary neurons stained by the antibody against β-III-tubulin and: (**b**) nicotinic acetylcholine receptor (nAChR); (**c**) synaptophysin; (**d**) NAT8L. The images are representative for four independent sets of experiments.

In these studies, we used two primary brain cell lines isolated from E18 embryos (Wistar rats). Primary neuronal culture was used to analyze the biochemical profile of neurons cultured without glial cells. Meanwhile, neural stem cells (NSC) provided the authors with data about the *N*-acetylaspartate network resulting from interactions between neurons and glial cells (cell types recognized in NSC: 70–80% astrocytes, 20–25% neurons, and up to 5% oligodendrocytes) (Figure 2a) [19].

**Figure 3:**
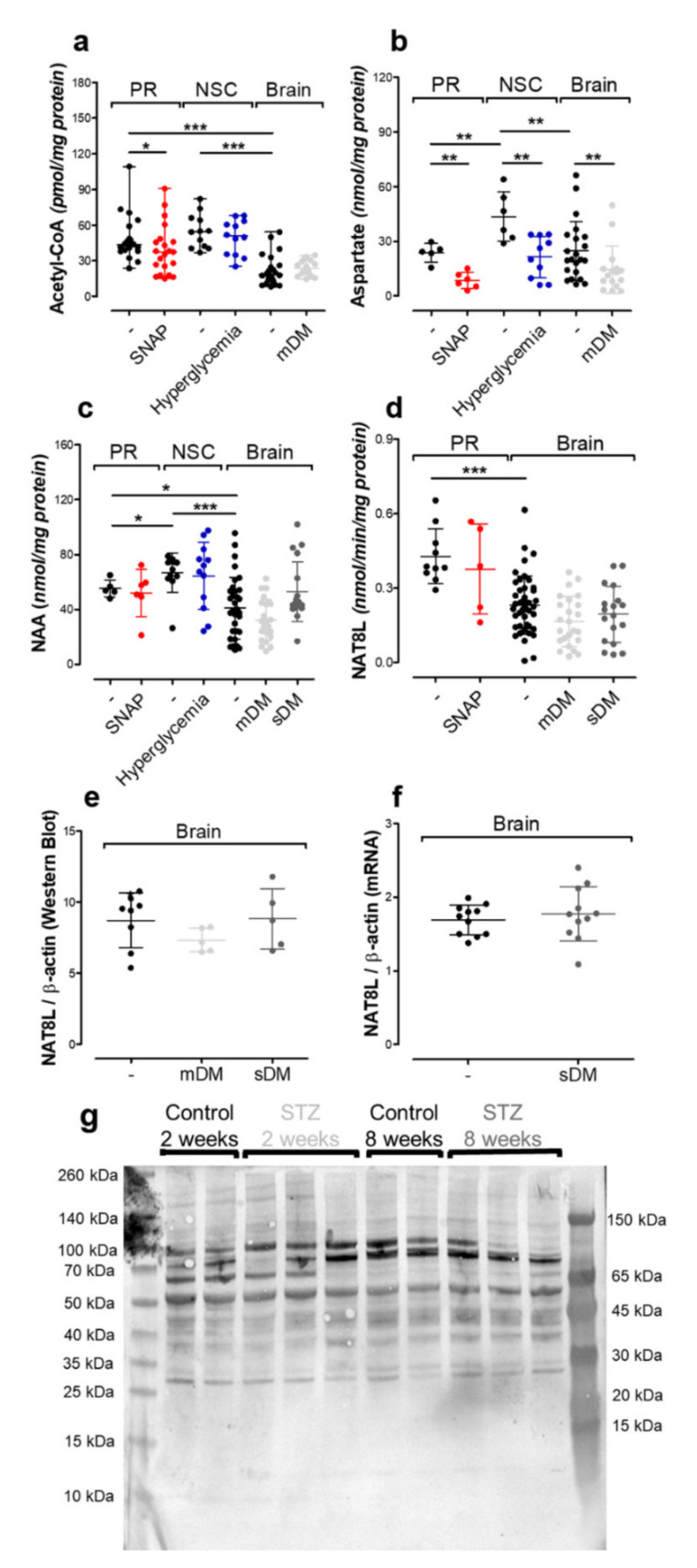
The impact of toxic factors on the NAA network measured in primary neurons (PR), neural stem cells (NSC) and brain tissue (Brain): (**a**) acetyl-CoA level; (**b**) aspartate level; (**c**) *N*-acetylaspartate level; (**d**) aspartate *N*-acetyltransferase (NAT8L) activity; (**e**) NAT8L protein level (Western blot); (**f**) *Nat8l* mRNA level (real-time RT-qPCR); (**g**) representative WB (Western Blot) membrane image for NAT8L protein. Data are means ± SD from 5–22 observations per group. Significantly different from the control: * (*p*–value < 0.05), ** (*p*–value < 0.01), *** (*p*–value < 0.001).

**Figure 4:**
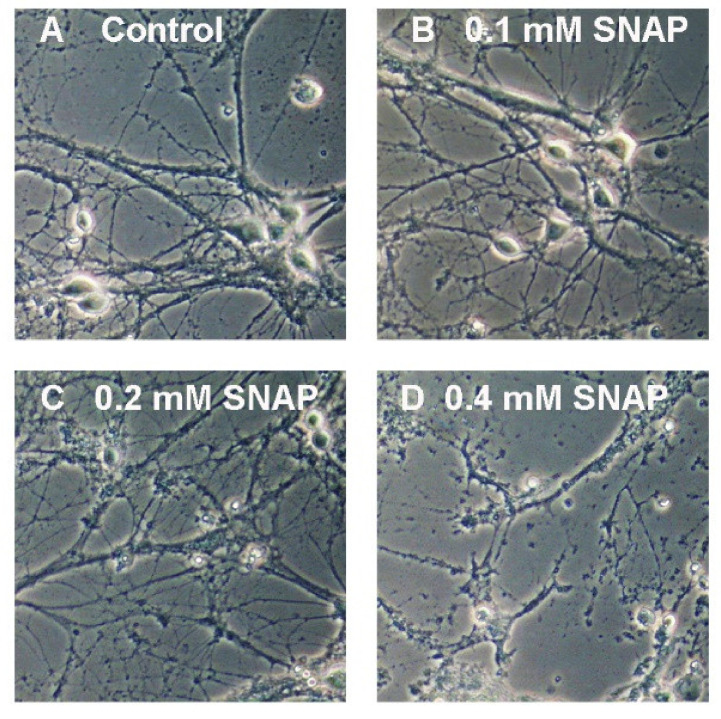
The impact of neurotoxic factors on primary neurons morphology or the *N*-acetylaspartate network related to neuronal energy pathways. The impact of SNAP: (**a**) control; (**b**) 0.1 mM SNAP; (**c**) 0.2 mM SNAP (in further experiments, used as basic SNAP concentration); (**d**) 0.4 mM SNAP.

Primary neurons should express a neuronal phenotype in terms of their characteristic morphology as well as higher glucose turnover when compared to glial cells [20,21,22]. To reach this phenotype, primary cells were allowed to mature for two weeks in neurobasal media supplemented with nerve growth factor (Materials and methods). Next, we analyzed the reaction carried out by the pyruvate dehydrogenase complex (PDHC), which introduces pyruvate and coenzyme A (CoA) to produce acetyl-CoA (Figure 1) [23]. Consequently, acetyl-CoA enters the tricarboxylic acid cycle and initiates the oxidative phosphorylation metabolism pathway (Figure 1) [23,24]. Alternatively, pyruvate can be consumed as a source either for oxaloacetate or lactate (Figure 1). Our data showed that primary neurons had significantly higher PDHC activity than neural stem cells (17.7 and 7.0 nmol/min/mg protein, respectively) (*p* < 0.05) (Table 1). Furthermore, primary neurons (PR), compared to neural stem cells (NSC), have a higher intracellular pyruvate concentration together with an equal acetyl-CoA level (Table 2 and Figure 3a). This indicates that our culture approach provides the PR cells with a high glucose turnover, resulting in a high pyruvate level (Table 2). Meanwhile, an unchanged acetyl-CoA level in primary neurons is linked with high acetyl-CoA consumption in the tricarboxylic acid cycle. Such consumption is reflected here by significantly higher citrate and oxaloacetate levels as well as significantly higher aconitase activity (*p* < 0.01, *p* < 0.05, and *p* < 0.05, respectively) (Table 1 and Table 2 and Figure 3a). With this primary neuronal culture approach, the primary neurons develop a strong neuronal network with a typical “neuron-like” morphology (Figure 4a).

### 2.2. Nitric Oxide Affects Primary Neurons Morphology and Energy State

**Figure 5:**
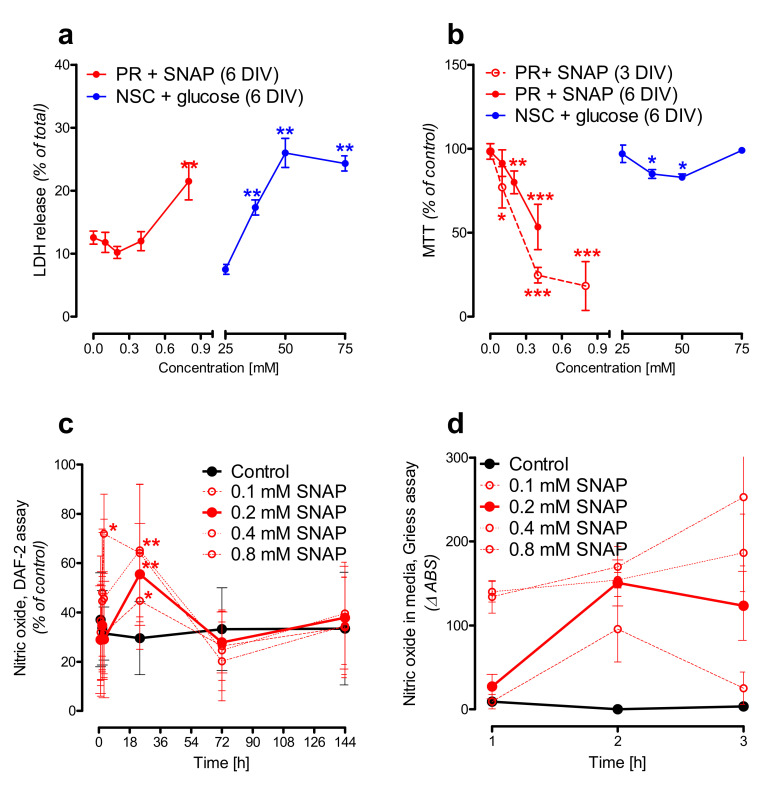
The impact of toxic factors on primary cells. (**a**,**b**) viability tests in both primary cell lines; (**c**,**d**) nitric oxide accumulation in primary neurons (PR). (**a**) LDH in media test; (**b**) methylthiazolyldiphenyl-tetrazolium bromide (MTT) test; (**c**) intracellular accumulation of nitric oxide (DAF-2 fluorescence assay); (**d**) in media accumulation of nitric oxide (Griess spectrophotometric assay). Data are means ± SD from 3 to 20 observations per group. Significantly different from the control: * (*p*–value < 0.05), ** (*p*–value < 0.01), *** (*p*–value < 0.001).

Our previous study showed that the 24 h treatment with neurotoxic Zn^2+^ concentrations resulted in a severe reduction of the *N*-acetylaspartate (NAA) level in SN56 neuroblastoma cells (the cellular model of cholinergic neurons) [9,25]. Further studies confirmed the significant reduction of acetyl-CoA (NAA substrate) and the suppression of NAT8L (an enzyme producing NAA) activity as well [9]. In our other studies, we noted that Zn^2+^-dependent NAT8L inhibition may be caused either by direct unspecific enzyme inhibition or by the promotion of oxidative stress affecting NAT8L activity. Therefore, considering these studies as well as the experimental studies showing that hyperglycemia may enhance NO production [26,27,28,29], we decided to expose primary neurons to the excess nitric oxide concentrations. SNAP (*S*-nitroso-*N*-acetylpenicillamine) is a commonly known nitric oxide (NO) generator releasing nitric oxide to culture media during the first hours of treatment [30]. In our studies, we used this generator to check whether SNAP-triggered neurodegeneration would affect *N*-acetylaspartate network homeostasis. Toxic SNAP concentration was established empirically starting from the 24 h treatment followed by 6 days of post-treatment culture (Materials and Methods). These 6 additional days of culture were added to analyze the reaction of stressed primary neurons. In terms of their morphology, 0.1 mM SNAP did not affect neuronal morphology (neither cell bodies nor neuronal networks were affected) (Figure 4b). Two times higher SNAP concentration (0.2 mM) dramatically affected the neuronal cell bodies, although to reach a visible disconnection of the neuronal network, we had to use SNAP in a concentration of at least 0.4 mM (Figure 4c,d). Using immunostaining against either the nicotinic acetylcholine receptor (nAChR) or synaptophysin, we learnt that 0.2 mM SNAP affected the cholinergic neurotransmission as well (Figure 2b,c). To connect nitric oxide toxicity with energy metabolism and the NAA network, the cell viability tests were measured. Lactate dehydrogenase (LDH) in media activity is well known as a membrane integrity test, while the methylthiazolyldiphenyl-tetrazolium bromide (MTT) test is an indicator for mitochondrial health. Each of the tested SNAP concentrations caused severe energy disruption, although only 0.8 mM SNAP caused a significant leakage of LDH enzyme to the culture media (*p* < 0.01) (Figure 5a). Furthermore, comparing 3 days and 6 days of post-treatment experimental time points, we observed that the energy shortages measured by the MTT test might have been partially reversible (Figure 5b). All these findings are consistent with the literature [30]. Next, we analyzed nitric oxide (NO) levels in both media (extracellular) and cells (intracellular). Since NO has a very short half-life, we assayed secondary NO metabolites, assuming them to be indirect indicators of NO-generation efficiency and cellular influx rate. Thus, we used a Griess assay to test nitrite levels in the media, while DAF-2 (4,5-diaminofluorescein diacetate) dyes were used to test the oxidation product of nitric oxide. To make the results less complex to analyze, they are reported as NO levels (Figure 5c,d). In the culture media, 0.2 mM SNAP reached the highest efficiency in NO production during the first 3 h of treatment, while cellular accumulation reached a plateau point after 24 h of treatment (Figure 5c,d). Compared to 0.2 mM SNAP, 0.8 mM SNAP generated two times more nitric oxide molecules in the media, although intracellular nitric oxide level was higher only by about 25%. However, since 0.8 mM SNAP disintegrates neuronal membranes, such concentration might artificially upregulate the intracellular NO level (Figure 4d). Then, 24 h exposure to 0.4 and 0.8 mM SNAP with an additional 6 days of culture resulted in considerable changes in the primary neuronal cells’ morphology, occurring together with severe LDH leakage as well as a more than 50% reduction in MTT level (Figure 4a–d and Figure 5a–d). Here, we assumed that these concentrations are lethal for the primary neuronal culture; therefore, in further studies, we used 0.2 mM SNAP.

**Figure 6:**
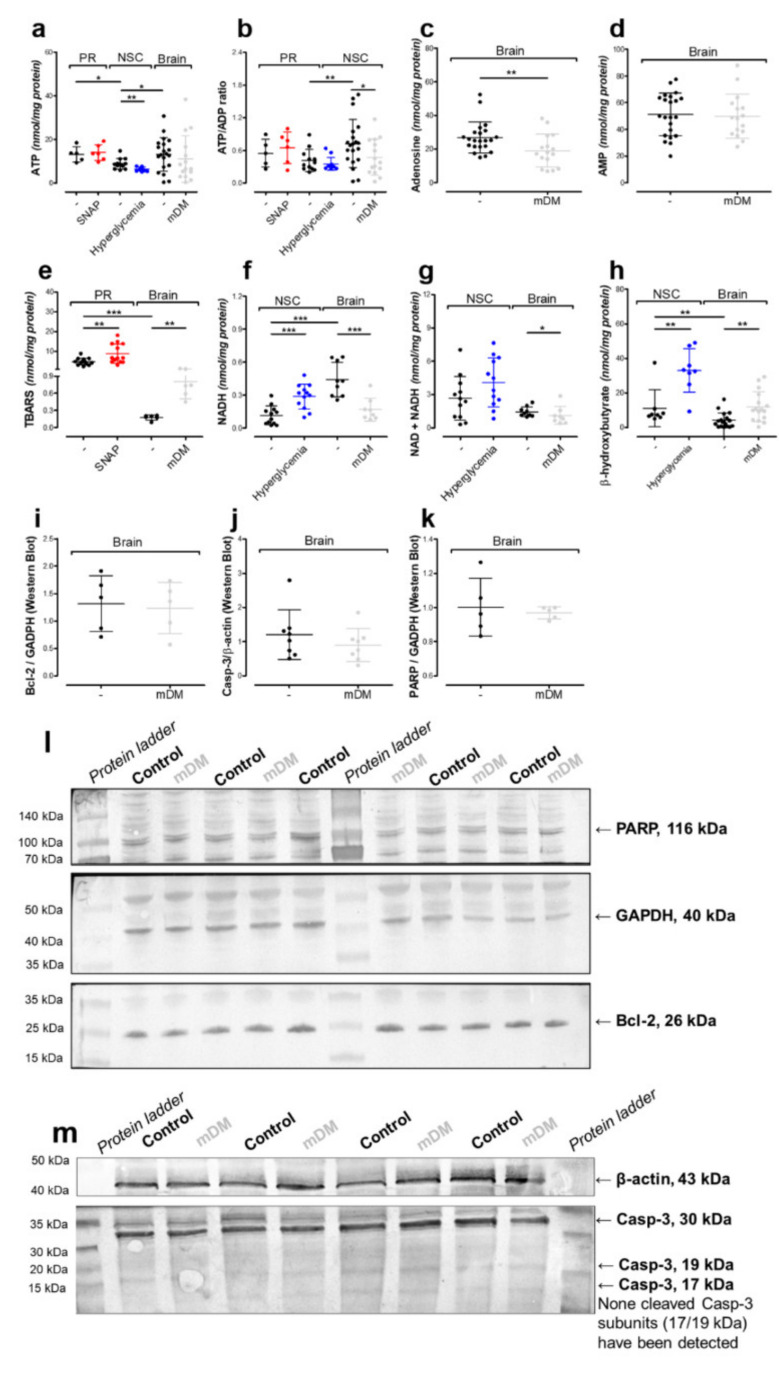
The impact of the toxic factors on intracellular homeostasis measured in primary neurons (PR), neural stem cells (NSC) and brain tissues (Brain). Nucleotides: (**a**) ATP level; (**b**) ATP/ADP ratio; (**c**) adenosine level; (**d**) AMP level; (**f**) NADH (reduced nicotinamide adenine dinucleotide) level; (**g**) total NAD (nicotinamide adenine dinucleotide) level. (**e**) TBARS level, cytosolic oxidative stress markers. (**h**) β-hydroxybutyrate level, ketoacidosis markers. Apoptosis markers (protein level measured by Western blot): (**i**) B cell lymphoma 2 (Bcl-2); (**j**) caspase-3 and cleaved caspase-3 (Casp-3); (**k**) poly-(ADP-ribose)-polymerase (PARP). Representative WB membrane images for: (**l**) PARP, Bcl-2, and GAPDH (glyceraldehyde 3-phosphate dehydrogenase) proteins; (**m**) Casp-3, cleaved Casp-3, and β-actin proteins. Data are means ± SD from 3 to 22 observations per group. Significantly different from the control: * (*p*–value < 0.05), ** (*p*–value < 0.01), *** (*p*–value < 0.001).

The accumulation of free radicals is the most common effect of excessive nitric oxide production, which we noted as a significant upregulation of thiobarbituric acid reactive substances (TBARS) level (a marker of cytoplasmic oxidative stress) (*p* < 0.01), although mitochondrial stress markers remained unchanged (aconitase, isocitrate dehydrogenase) (Table 1 and Figure 6e) [30]. The nitric oxide-triggered cytoplasmic disruptions came with severe outcomes regarding the levels of metabolites (Table 2 and Figure 1 and Figure 3a,b). Recently, NO has been reported to inhibit the pyruvate dehydrogenase activity [31,32]. We hypothesize that excess nitric oxide inhibits pyruvate dehydrogenase complex activity, which slows down the tricarboxylic acid cycle and malate–aspartate shuttle (diminished levels of citrate, oxaloacetate, acetyl-CoA, and aspartate) (Table 1 and Table 2 and Figure 1 and Figure 3a). However, since our method measure enzyme activity in adaptive conditions, we did not note any changes in pyruvate dehydrogenase activity (Table 1). In order to keep stable the ATP flux, energy metabolism has been partially rerouted to LDH-dependent pathway vigorously consumed pyruvate resulting with the significant elevation of the lactate level (*p* < 0.001) (Table 1 and Table 2) [22,23]. Described impairment reflects metabolic disturbances under diabetic ketoacidosis; therefore, these conditions have been used to analyze the NAA network further in this study.

### 2.3. Hyperglycemia Affects Neural Stem Cells Mitochondrial Homeostasis

In research on diabetes, discussion has increasingly become focused on the neuropathology created by unstable glycemia in blood cells involved in the vascular system surrounding the brain cells [33]. Under physiological circumstances, glucose is transported from the bloodstream to brain cells via blood–brain barrier glucose-specific transporters, predominantly GLUT1. Uncontrolled hyperglycemia could dramatically increase the neuronal glucose level, which leads to neuronal damage, known as glucose neurotoxicity [33]. Since glucose-derived energy metabolism and the *N*-acetylaspartate network (NAA network) are closely connected with each other, we consider hyperglycemia as one of the potential risk factors for NAA network stability (Figure 1). To conduct energy metabolism, neurons are fed by astrocytes through neuronal-astrocyte coupling with glucose and lactate [34]. Therefore, the impact of hyperglycemia on the NAA network has been studied using neural stem cells (NSC) instead of primary neurons. Here, we used a widely known in vitro model of hyperglycemia with a daily change of media [35]. The final glucose concentration in the culture media ranged between 25 and 75 mM (Materials and methods).

The LDH test showed glucose-dependent membrane disruption (Figure 5a), although NSC morphology did not support these findings (data not shown). Such an unusual phenomenon arose from the upregulation of LDH activity in NSC cells cultured in hyperglycemic conditions (Table 1). In the control conditions (NSC cells cultured in media with 25 mM glucose), the LDH cellular activity was 0.4 µmol/min/mg protein, while LDH activity in media was less than 10% (Table 2 and Figure 5a). The 50 mM glucose concentration increased LDH activity by almost 30% in NSC cells and three times in its culture media (Table 1 and Figure 5a). In both glucose treatment approaches, each time, the maximal/total/100% LDH in media activity was established using NSC cultured in media with 25 mM glucose (Supplement 1). Ultimately, the LDH in media test did not consider the impact of elevated LDH activity in the NSC cells. Therefore, the same degree of LDH leakage from NSC cells treated with 25 mM and 50 mM glucose ended up with significantly higher LDH in media activity in NSC cells treated with 50 mM glucose (*p* < 0.01) (Table 1 and Figure 5a,b). The MTT test indicated 37.5 mM and 50 mM glucose as the glucose concentrations evoking disruption in NSC mitochondrial energy metabolism (Figure 5b).

Astrocytes are known to be cells, which prefer the LDH/lactate-dependent energy production pathway instead of the tricarboxylic acid cycle [36,37]. Additionally, they are the main cell type in the NSC culture, although the biochemical profile of NSC arises from all the cell types that are in this culture [19,37]. Therefore, in the NSC culture, we were able to observe changes in the lactate/pyruvate ratio resulting from interactions between glial cells and neurons (Table 2). The lactate/pyruvate ratio is especially important in diabetes. In clinical practice, the most dangerous diabetes side effect is ketoacidosis along with an increased NADH/NAD ratio (reduced nicotinamide adenine dinucleotide / nicotinamide adenine dinucleotide ratio), which occurred in our culture system as well (Figure 6f,h). Many studies have shown that astrocytes are the only possible source of the ketone bodies produced in the brain [38,39,40]. Overproduced β-hydroxybutyrate can serve as an alternative source for neuronal acetyl-CoA production; therefore, no reduction of acetyl-CoA was noted in our studies (Figure 3a and Figure 6h) [36,37]. In hyperglycemic conditions, pyruvate is overproduced in glycolysis. Next, the overproduced pyruvate either undergoes reduction by overactive LDH to lactate or enters the mitochondria to initiate the tricarboxylic acid cycle via overactive synthase citrate (Table 1 and Table 2 and Figure 1) [41]. Excessive glycolysis and overactive tricarboxylic acid cycle pathways favor NAD turnover to NADH, increasing the NADH level without changes in total NAD + NADH level (Figure 6f,g). In addition, it has been reported that diabetes-related disturbances in NADH/NAD ratio might be related to an overactivation of the poly-(ADP-ribose)-polymerase (PARP) enzyme, which use NAD as a substrate [42]. However, our data showed no upregulation in the PARP protein level (Figure 6k,l).

Hyperglycemia triggered neurons to overproduce pyruvate, which is catalyzed by pyruvate dehydrogenase complex, which in theory should accelerate tricarboxylic acid turnover. However, an increased NADH/NAD ratio is an activation factor for pyruvate dehydrogenase kinase, which by the phosphorylation of PDHC prevents its over-activation (Table 1 and Figure 6f,g) [43]. As stated previously, the struggle of the ketoacidosis outcome with neurons resulted in acetyl-CoA production from an alternative pathway using β-hydroxybutyrate as a substrate; therefore, no reduction of acetyl-CoA was reported in our studies (Figure 3a and Figure 6h). The overall conclusion arising from this part of the study is that the cells kept in the presence of high concentrations of glucose will have impaired energy metabolism and, probably, perturbed astrocyte–neuron coupling. This would make the model suitable for studies of the NAA network and its metabolic fragility.

### 2.4. In Vivo Model of Hyperglycemia

**Figure 7:**
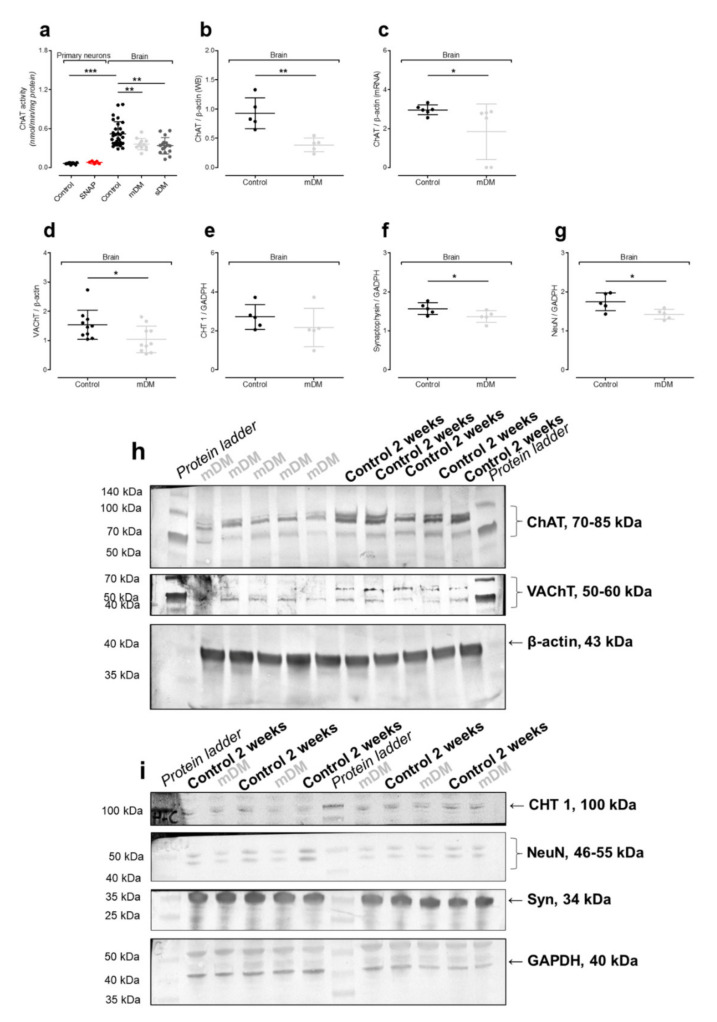
The impact of toxic factors on the neurotransmission markers measured in the primary neurons (PR) or the rats’ brain tissues (Brain). (**a**) ChAT activity; (**b**) choline acetyltransferase (ChAT) protein level (Western blot); (**c**) *Chat* mRNA level (real-time RT-qPCR); (**d**) vesicular acetylcholine transporter (VAChT) protein level (Western blot); (**e**) high-affinity choline transporter (CHT 1) protein level (Western blot); (**f**) synaptophysin (Syn) protein level (Western blot); (**g**) NeuN protein level (Western blot); (**h**) representative WB membrane images for ChAT, VAChT, and β-actin proteins; (**i**) representative WB membrane images for CHT 1, NeuN, Syn, and GAPDH proteins. Data are means ± SD from 3 to 16 observations per group. Significantly different from the control: * (*p*–value < 0.05), ** (*p*–value < 0.01), *** (*p*–value < 0.001).

Our previous studies revealed that inside the cholinergic neurons, *N*-acetylaspartate production is closely related to cholinergic neurotransmission [9]. On the other hand, diabetes has been reported as a disease affecting cholinergic neurotransmission via the downregulation of cholinergic acetyltransferase [44]. Therefore, both lines were tested for choline acetyltransferase (ChAT) activity, although ChAT activity remained undetectable (NSC, data not shown) or too low to notice any important changes (primary neurons) (Figure 7a). Since our in vitro models were not sufficient for cholinergic neurotransmission studies, we analyzed the diabetic Wistar rat brains (Materials and methods). The animals had increased brain hexokinase activity, significant body weight loss, polyuria, and urinal hyperglycemia with ketoacidosis (*p* < 0.001) (Table 1 and Table 3), which is consistent with other researchers’ reports [45].

In cholinergic neurons, choline acetyltransferase (ChAT) introduces choline to acetyl-CoA in order to produce acetylcholine [46]. To reach the synaptic cleft, acetylcholine is carried by the vesicular acetylcholine transporter (VAChT) [46]. During ongoing neurotransmission, released acetylcholine interacts with the acetylcholine receptor co-localized with acetylcholine esterase [46]. Esterase cleaves the neurotransmitter to choline and acetate. Finally, choline returns to cholinergic neurons via the high affinity choline transporter (CHT 1) [46]. ChAT activity was significantly affected in both experimental time points (2 and 8 weeks as well) by the hyperglycemia-dependent downregulation of *Chat* gene expression (*p* < 0.01, *p* < 0.01, *p* < 0.01, respectively) (Figure 7a–c,h). This downregulation resulted in lower vesicular acetylcholine transporter (VAChT) protein levels as well (Figure 7d,h). This suggested that streptozotocin (STZ)-triggered hyperglycemia resulted in cholinergic neurons being disabled to provide fully efficient choline neurotransmission. We learned that neither the downregulation of ChAT nor VAChT affected the high-affinity choline transporter (CHT 1) protein level (Figure 7e,i). Studies with VAChT-KD^H^°^M^ transgenic mice having Vacht deficiency showed that the downregulation of VAChT does not affect either acetylcholine esterase, the muscarinic acetylcholine receptor 2, or CHT 1, either [47]. Due to the lack of the molecular studies dealing with the CHT 1 protein, this phenomenon still does not have a clear explanation. We might only assume that diminished capacity to provide cholinergic neurotransmission is not a trigger snap to modify expression of the *Slc5a7* gene encoding CHT 1 (Figure 7e,i). Moreover, in the course of diabetes, apart from poor cholinergic neurotransmission, the streptozotocin-affected brains have to deal with a decreased number of NeuN (+) and synaptophysin (+) neurons, which do not upregulate the apoptotic processes (Figure 6f,g,i,j). Our findings indicate that the downregulation of cholinergic neurotransmission without cell loss is consistent with the literature [44,48,49]. Unfortunately, no good molecular explanation has been found to fit the data. It seemed that insulin-dependent protein kinase A changes gene expression such in a way so that cholinergic markers or even neuronal maturation processes might be suppressed [44,48,49].

In brain tissue, increased β-hydroxybutyrate and lactate levels confirmed ongoing lactic acidosis with ketoacidosis, while increased TBARS level as well as unchanged aconitase and isocitrate dehydrogenase activities indicated ongoing cytoplasmic oxidative stress, as well (Table 1 and Figure 6e,h). Chronic, severe hyperglycemia did not upregulate PARP protein level (the above-mentioned enzyme converts NAD to NADH) or apoptotic markers, such as cleaved caspase-3 (Casp-3) or B cell lymphoma 2 (Bcl-2) (Figure 6i–m). Considering the influence of ketoacidosis on PDHC and LDH activities as well as acetyl-CoA and pyruvate levels, we noted that chronic hyperglycemia with acidosis did not support pyruvate accumulation. However, overproduced β-hydroxybutyrate prevents acetyl-CoA shortages (Table 1 and Table 2 and Figure 3a and Figure 6h). The 2-week-long hyperglycemia resulted in lactic acidosis, decreasing ATP levels (Table 2 and Figure 6a).

In the brain, there is no de novo synthesis pathway for adenosine [50]. It has been shown that here, adenosine production enters the synaptic cleft from ATP produced and released by astrocytes [50]. Therefore, the ATP reduction suppressed the adenosine level as well, although other adenosine-based nucleotides remained unchanged (Figure 6a–d). The 6-day-long hyperglycemia analyzed using the NSC cells indicated a dysregulation in cellular homeostasis between NAD and NADH (Figure 6f,g). Hyperglycemia lasting for 14 days (2 weeks) in the rats’ brains considerably decreased the total level of nicotinamide adenine dinucleotides, which subsequently led to a reduction of NADH level as well (Figure 6f,g). All these findings are consistent with other researchers’ reports [51,52]. NSC cells reacted differently to glucose elevation than Wistar rat brains. In both models, the pyruvate dehydrogenase activity remained unchanged. In NSC cells, ketoacidosis increased pyruvate level, probably via pH-dependent LDH inhibition. Consequently, increased pyruvate levels stimulated citrate synthase activity, which overproduced citrate. These results suggest that NSC cells prioritized oxidative phosphorylation over anaerobic metabolism. In the Wistar rat brain, lactic acidosis and ketoacidosis did not change the mentioned parameter, which might indicate the development of anaerobic metabolism. In conclusion, these two models might reflect the different impact of hyperglycemia on the NAA network. Moreover, the in vivo model introduces the impact of cholinergic neurotransmission (and choline acetyltransferase downregulation) to the studies with NAA network fragility.

### 2.5. N-Acetylaspartate Network is Resistant to NO- and Hyperglycemia-Derived Toxicity

The *N*-acetylaspartate network (NAA network) begins inside the neurons and then ends up either in oligodendrocyte-derived myelin or in astroglial energy pathways [2,8,13,53,54,55,56]. Such a complicated molecular machinery is entirely controlled by one single enzyme called aspartate *N*-acetyltransferase (NAT8L, the Shati protein), which seems to be affected by dementia-related neurodegeneration processes [53,57,58,59,60,61]. Researchers working with *Nat8l/Shati*-/- knock-out mice have indicated that NAT8L might control our emotions, and that the *Nat8l* gene mutation promotes autism-like behavior or provokes the tendency to develop adult depression [2,13,55,56,57,58,59,60,61]. Moreover, clinical studies showed that maternal diabetes might increase a risk of autism spectrum disorders, which links hyperglycemia with the NAA network [62].

In the primary neuronal culture treated by exogenous nitric oxide, acetyl-CoA and aspartate levels were significantly affected (*p* < 0.05 and *p* < 0.01, respectively), although the NAA level remained constant, along with NAT8L level/activity as well as NAT8L subcellular localization (Figure 3a–g). Hyperglycemia-derived acidosis together with oxidative stress jointly have been studied as a potential NAA network suppressor in neural stem cells. This time, we used an approach in which toxicity needs time to build up a noticeable pathology. Since the astroglia feed the neurons with glucose and lactate, in hyperglycemic conditions, instead of protecting neurons, astrocytes intensified neuronal pathology [34]. This time, acetyl-CoA and *N*-acetylaspartate levels were not affected, although the level of aspartate was considerably decreased (Figure 5a–c).

Finally, the in vivo model was used to reflect interactions between all cell types in the central nerve system (including cholinergic neurons). Two weeks after STZ injection was enough time to build up hyperglycemia-triggered toxicity affecting cholinergic neurotransmission (Figure 7a–g). To compare the impact of shorter and longer effects of ketoacidosis on the NAA network, we extended the experimental outline for an additional 6 weeks (8 weeks together). Each time, we noted severe energy disruption going on in the animals’ brains, although the *N*-acetylaspartate level was resistant to this pathology (Figure 3c). Moreover, our data showed that neither NAT8L activity, *Nat8l* mRNA level, nor protein level were affected by STZ-induced hyperglycemia (Figure 3c–g).

## 3. Materials and Methods

### 3.1. Materials

Unless specified otherwise, all the used compounds were specified in Table S1, while cell culture disposables were provided by Sarstedt (Blizne, Łaszczyńskiego, Poland). Unless specified otherwise, spectrophotometric assays were run either using an Ultraspec 3100 Pro (Amersham Biosciences, Amersham, UK) or, for multiple well plates-based assays, a Victor 3, 1420 Multilabel Counter (Perkin Elmer, Warsaw, Poland).

### 3.2. Ethics Approval

All experiments were approved by the Polish Bioethics Committee (44/2016, 23 November 2016, Bydgoszcz, Poland). Studies followed the EU Directive 2010/63/EU and the International Council for Laboratory Animal Science (ICLAS) guidelines for animal experiments.

### 3.3. Animals (In Vivo Studies)

White male adult Wistar rats were housed at the Animal House (Medical University of Gdansk, Gdansk, Poland) with access to food and water ad libitum under a standard 12 h light/12 h dark cycle. The rats’ average weight before the experiments was 180–230 g followed by a weight of 200–350 g at the end of the experiments.

For the purposes of this study, the animals were divided randomly into different treatment groups with the following group size: sham control group—17 male rats, moderate diabetes mellitus group—8 male rats, severe diabetes mellitus group—6 male rats.

Diabetes mellitus (DM) was induced by a single intraperitoneal injection of 65 mg/kg b.w. streptozotocin (STZ) in 0.1 M citrate buffer (ip). The animals were sacrificed 2 weeks (moderate DM, mDM) or 8 weeks (severe DM, sDM) after injection. The sham control group was treated with a similar volume of buffer without streptozotocin.

Glycemia was measured on the third day after streptozotocin administration by serum glucose level measurement (Accu-CHEK Performa glucometer kit, Roche, Warsaw, Poland). Animals with a blood glucose ≥ 17 mM or greater were deemed diabetic and suitable for this study (Table 3) [63]. For the last 24 h of in vivo experiments, the animals were transferred to a metabolic cage and 24 h urine samples were collected (Table 3). Before the animals were euthanized by pentobarbitone overdose (2 mL/kg b.w., ip, concentration: 0.66 M), blood glucose level was determined (as described above).

### 3.4. Primary Cultures (In Vitro Studies)

Embryonal brain cortices (E18) obtained from pregnant Wistar rats were dissected in Hank’s buffered salt solution (HBSS) supplemented by 8 mM HEPES (pH = 7.4), 100 U/mL penicillin, and streptomycin. Using different culture strategies, the cells were settled for either primary neuronal culture (PR) or neural stem cells culture (NSC) [19,64].

### 3.5. Embryonic Primary Neurons (PR)

Isolated cells were seeded as a monolayer in poly-l-ornithine and laminin-coated dishes. In order to obtain mature neurons, the cells were cultured for 14 days in a neurobasal medium supplemented with B27 supplement, 2 mM Glutamax, 10 ng/mL nerve growth factor (NGF), 20 nM AraC, and 100 U/mL penicillin with streptomycin. Every third day of the culture, the media were changed in half of their volume. For further experiments, culture dishes were assigned randomly to different treatment experimental time points. Mature primary neurons were cultured in the same medium, although in order to study the impact of oxidative stress on the cells, the B27 supplement was changed to B27 minus antioxidant supplement. For 24 h after maturation, the primary neurons were cultured in the presence of 0–0.8 mM S-nitroso-N-acetylpenicillamine (SNAP). On the following day, the media were changed to fresh ones without SNAP and the cells were maintained in culture for an additional three to six days in vitro (DIV) [64].

### 3.6. Embryonic Neural Stem Cells (NSC)

Dissected cortices were cultured as neurospheres in DMEM/F12–GlutaMAX media supplemented with B27 supplement, 10 ng/mL basic fibroblast growth factor, 20 ng/mL epidermal growth factor, and 100 U/mL penicillin with streptomycin. Neurospheres were passaged every third day of culture. Cells between 1 and 3 passages were considered as suitable for this study. Neural stem cell types were recognized by β-III-tubulin (neurons), glial fibrillary acidic protein (GFAP) (astrocytes), and CNPase (2′,3′-Cyclic-nucleotide 3′-phosphodiesterase, oligodendrocytes) specific markers (Table S2). For the experimental step, neural stem cells (NSC) were seeded as a monolayer in poly-l-ornithine and laminin-coated dishes and left for 24 h in neurosphere media. To initiate cell differentiation, the cells were cultured for another 6 days in media without growth factors. For further experiments, culture dishes were assigned randomly to different treatment experimental time points. Here, NSC cells grew in media with 25–75 mM glucose and supplement B27 minus antioxidants instead of the standard B27 supplement. In order to replenish the glucose level, the media were changed each day of the culture [19].

### 3.7. Sample Preparation

Brain tissues (without cerebellum) or cells were homogenized in chilled: 4% HClO4 (for metabolic studies); 0.1 M HCl (for NAD assay); 0.2 M KOH (for NADH assay); or 0.32 M sucrose buffered by 5 mM HEPES (pH = 7.4) with 0.1 mM EDTA (ethylenediaminetetraacetic acid, for enzymatic assays). After centrifugation at 13,000× *g* (4 °C, 15 min), each sample was immediately used for studies or kept at −80 °C until analyzed.

### 3.8. Morphology Imaging

Images were captured under 40× magnification using an inverted light microscope (Axiovert 25, Zeiss) [9].

### 3.9. Enzymatic Assays

To analyze enzymatic activity in the cell lines, from each dish, two independent cell lysates were collected and reported as one average result. To analyze enzymatic activity in brain tissue, 3 tissue lysates per one brain were collected, and then, the enzymatic profile of each lysate was measured in two independent samples. Eventually, 3 average results per 1 brain were reported in this study. Protocols for enzymatic assays were described with details in our previous study [25].

Aconitase (Aco, EC 4.2.1.3) [25], aspartate aminotransferase (GOT, EC 2.6.1.1) [25], aspartate *N*-acetyltransferase (NAT8L, EC 2.3.1.17) [25], citrate synthase (SC, EC 2.3.3.1) [25], choline acetyltransferase (ChAT, EC 2.3.1.6) [25], isocitrate dehydrogenase (IDH, EC 1.1.1.42) [25], pyruvate dehydrogenase complex (PDHC, EC 1.2.4.1) [25].

Hexokinase (Hex, EC 2.7.1.1) activity was determined using the NADPH/NADP conversion technique at λ = 340 nm and 37 °C. Then, 1 mL of reaction buffer contained 0.1 M Tris-HCl (pH = 7.4), 5 mM glucose, 0.2 mM MgCl_2_, 0.25 mM NADH, 1 U glucose-6-phosphate dehydrogenase (G-6-PDH, EC 1.1.1.49), and 20 μg of cell homogenate protein. Enzymatic assay was initiated by the addition of 10 μL of 0.1 M ATP [65].

Lactate dehydrogenase or LDH in media assay (LDH, EC 1.1.1.27) activity was determined using the NADH/NAD conversion technique, at λ = 340 nm and 37 °C. Then, 1 mL of reaction buffer contained 0.1 M Tris-HCl (pH = 7.4), 0.2 mM NADH, and 20 μg of cell homogenate protein (or 0–200 μL of culture media, for cell viability test assay). Enzymatic assay was initiated by the addition of 10 μL of 0.1 M pyruvate [66].

Pyruvate dehydrogenase complex (PDHC, EC 1.2.4.1.) activity was determined using cycling method. In each lysate (100 μg of cell homogenate protein), the following reactions were performed: (1) citrate production and (2) citrate level measurement. The first reaction (1) was carried out in 250 μL for 30 min (37 °C, gentle shaking), the buffer contained 0.1 M Tris-HCl (pH = 8.3), 2 mM MgCl_2_, 10 mM dithiothreitol, 10 mM pyruvate, 2 mM thiamine pyrophosphate, 0.2 mM CoA, 2.5 mM oxaloacetate, 2 mM NAD, and 0.15 U citrate synthase (EC 4.1.3.7). Reaction was terminated by thermic shock (10 min, 100 °C). Finally, the produced citrate level was determined using NADH/NAD conversion technique at λ = 340 nm and 37 °C. The reaction buffer contained 0.1 M Tris-HCl (pH = 7.4), 0.1 mM NADH, 0.2 U malate dehydrogenase (EC 1.1.1.37), and 100 μL of achieved supernatant in a final volume of 0.7 mL. The assay was initiated by the addition of 10 μL of 0.1 U citrate lyase (EC 4.1.3.6). The blank sample was treated in the same way as the measured sample, but the blank sample was not reached in CoA [67].

### 3.10. Metabolic Assays

To analyze metabolic profiles, we collected two independent acidic supernatants per each cell culture dish or three independent acidic supernatants per one rat brain. After 15 min of deproteinization (at 4 °C), samples were centrifuged at 13,000× *g* (4 °C, 15 min). Next, ice-cold samples were neutralized and immediately assayed.

Protocols for acetoacetate, acetyl-CoA, aspartate, β-hydroxybutyrate, lactate, *N*-acetylaspartate, oxaloacetate, pyruvate, and TBARS assays were described with details in our previous study [25].

Protocols for ATP and ADP, AMP, adenosine [68], citrate [67], the MTT viability test [41], nitric oxide (DAF-2 staining) [69], Griess assay [70]), NAD, and NADH [71] assays are described with details in Supplement 1.

### 3.11. Real-Time RT-qPCR Analysis of Nat8l and Chat mRNA Levels

The 2 × 10^6^ cells or 0.1 g of brain tissues were vortexed or homogenized in a sterile tube with 0.5 mL (cells) or 1 mL (brain tissue) of RNA Extracol extraction buffer (Eurx, Cat #E3700-02, Gdansk, Poland). The extraction was initiated by the addition of 250 μL chloroform (per 1 mL of RNA Extracol buffer). After vigorous shaking, each sample was incubated at 4 °C for 15 min and spun down (10,000× *g* for 15 min at 4 °C). The upper aqueous phase was transferred to a new tube and refilled by isopropanol in a 1:2 ratio (isopropanol: RNA Extracol, *v*/*v*). The RNA precipitation was carried out overnight at −20 °C, and on the following day, each sample was centrifuged (10,000× *g* for 15 min at 4 °C). The RNA pellet was washed firstly with 99.8% and then with 75% ethanol, finally air-dried, and reconstituted in nuclease-free water (15–20 μL) (Sigma Aldrich, Cat# W4502, Poznań, Poland). The obtained samples were kept at −20 °C until analyzed. The quantity of isolated RNA was determined using the Qubit RNA HA assay kit according to the manufacturer’s instructions (ThermoFisher Sc., Cat #Q32855, Warsaw, Poland). The gene expression levels of *Nat8l* encoding the NAT8L enzyme and *Chat* encoding the ChAT enzyme were determined by real-time RT-qPCR performed in a Light Cycler 480 II (Roche Diagnostic GmbH, Penzberg, Germany) using a Path-IDTM Multiplex One-Step RT-PCR Kit (ThermoFisher Sc., Cat #4442135, Warsaw, Poland) and Universal ProbeLibrary for the rat species, and gene-specific intron-spanning primers (Table 4). The reaction mixture in the final volume 10 μL contained 5 μL of Multiplex RT-PCR Buffer, 1 μL of Multiplex Enzyme Mix, and 0.5 μL of each primer for the target transcript, 0.2 μL of a target probe, 0.2 μL of primers’ reference gene, 0.2 μL of a probe for the reference transcript, and 2 μL of total RNA (Table 4). The target gene transcript levels were normalized to the reference transcript of the β-actin gene (*Actb*). Reverse transcription program: 48 °C–10 min and 95 °C–10 min. Amplification program: 95 °C–15 s, 60 °C–45 s for 45 cycles. Data were processed with Light Cycler 480 II software 2.0 [25].

### 3.12. Double Staining in Immunocytochemistry

For immunocytochemistry, cells were seeded on cover slips and cultured as usual. Next, the cells were fixed with 4% paraformaldehyde in sterile PBS (pH = 7.4, 15 min, room temperature) followed by 3 washes with PBS. The fixed cells were left in PBS at 4 °C until needed. Before immunostaining, the cells were permeabilized for 30 min in PBS buffer with 0.1% Triton X-100 and 5% bovine serum albumin. The overnight incubation of cover slips was carried out in 0.1% Triton/0.5% bovine serum albumin/PBS and two primary antibodies diluted to 1:200 (*v*/*v*) with two different hosts (Table 5). On the following day, the cover slips were washed 3 times in PBS and then incubated for 3 h in PBS buffer with 0.1% Triton, 0.5% bovine serum albumin, and two Alexa Fluor^®^-conjugated secondary antibodies (1:500, *v*/*v*) (Table 5). Finally, the cover slips were incubated for 5 min with 0.2 µM DAPI (4′,6-diamidyno-2-fenyloindol) in PBS buffer with 0.1% Triton and 0.5% bovine serum albumin. After an additional 3 washes in PBS, the cover slips were mounted with ProLong^TM^ Glass Antifade Mountant (ThermoFisher Sc., Cat #P36980). Images were captured under 60x magnification using an inverted fluorescence microscope (Olympus IX83, Olympus) [72].

### 3.13. Western Blot Analysis

The brain tissue samples were lysed for 30 min in lysis buffer (1% protease inhibitor cocktail, 50 mM Tris-HCl buffer pH 7.4, 5 mM EDTA, 100 mM NaCl, 1% Triton-X100, 5% glycerol, 10 mM KH_2_PO_4_) at 4 °C. The obtained lysates were kept at −20 °C until analysis. Each sample (40 µg of protein/20 µL of 10 mM dithiothreitol/Laemmli buffer) was incubated for 1 h at 57 °C followed by the addition of 2 µL 0.5 M 2-chloroacetamide and incubation for an additional 1 h at room temperature. Next, the spun down samples (10,000× *g* for 2 min at 4 °C) were loaded to ExpressPlus^TM^ PAGE Gel 4–20% gradient BisTris-PAGE gels (GenSignal, Cat #GS1960, Warsaw, Poland). Then, the BisTris-PAGE gel was run at 300 V for 17 min in MOPS/SDS running buffer (1 M Tris pH = 7.7, 1 M MOPS, 70 mM SDS, 20.5 mM EDTA) in a MINI-PROTEAN electrophoresis system with cooling (Roche, Warsaw, Poland). Next, proteins were transferred from the Bis Tris-PAGE gel to a PVDF membrane (pore size: 0.2 µm, iBlot^®^ transfer stack, Cat #IB401001, Warsaw, Poland) using the iBlot^®^ Dry Blotting System with P0 program (program details: 1 min–20 V, 4 min–23 V, 3 min–25 V) (ThermoFisher Sc., Dreieich, Germany). The PVDF membrane was washed 2 × 10 min in TBTS buffer (25 mM Tris-HCl pH = 7.4, 135 mM NaCl, 3 mM KCl, 0.5% Tween20). Non-specific bindings were blocked with 5% BSA in TBST (60 min, room temperature). Next, the PVDF membrane was incubated with specific primary antibodies (1:500, *v*/*v*) in 5% BSA/TBST buffer (4 °C, overnight) (Table 5). On the following day, after 4 × 10 min washing, the membrane was incubated with polyclonal AP-conjugated secondary antibodies diluted 1:5000 (*v*/*v*) with 5% BSA/TBST buffer (3 h, room temperature) (Table 5). The PVDF membrane was developed in a dark room for 15 min with developing buffer (0.1 M Tris buffer pH = 9.5, 0.1 M NaCl, 5 mM MgCl_2_, 0.33 mg/mL nitrotetrazolium Blue chloride, and 0.17 mg/mL BCIP) [72].

### 3.14. Protein Assay

Protein was assayed as previously [25].

### 3.15. Statistics

The results are presented as a means ± standard deviation of the mean (SD). The Kolmogorov–Smirnov normality test excluded normal data distribution. Therefore, the results were tested by either the Mann–Whitney test or Kruskal–Wallis followed by Dunn’s Multiple Comparison post-test, where values of *p* < 0.05 were considered statistically significant. We performed all statistical analyses using the Graph Pad Prism 4.0 statistical package (Graph Pad Software, San Diego, CA, USA).

## 4. Conclusions

The *N*-acetylaspartate network is created by a reaction between acetyl-CoA and aspartate metabolites introduced by aspartate *N*-acetylaspartate (NAT8L), while cholinergic neurotransmission starts from the reaction between acetyl-CoA and choline catalyzed by choline acetyltransferase. Our data clearly show that type 1 diabetes-like conditions triggers disturbances in energy metabolism. Under these conditions, brain cells kept the *N*-acetylaspartate level and NAT8L activity stable, but cholinergic markers were downregulated. In our previous study, we showed that the progression of Alzheimer disease-associated cholinergic neurodegeneration accompanied the NAT8L inhibition. The same pattern was identified in type 1 diabetes patients. Thus, we conclude that these diseases share pathomechanisms that trigger *N*-acetylaspartate depletion by the inhibition of NAT8L gene expression or enzyme activity rather than by substrate shortages.

## Figures and Tables

**Table 1:** Enzymatic activities measured in Wistar rat brains.

Parameter	Added	Primary Neurons	Neural Stem Cells	Wistar Rat’s Brain Tissue
Pyruvate dehydrogenase complex nmol/min/mg protein	Control/Sham	17.7 ± 4.9	7.0 ± 3.0	23.8 ± 9.9
	0.2 mM SNAP	22.8 ± 6.8		
	50 mM glucose		6.2 ± 2.0	
	mDM			17.4 ± 4.9
Lactate dehydrogenase µmol/min/mg protein	Control/Sham		0.4 ± 0.1	1.3 ± 0.4
	50 mM glucose		0.5 ± 0.6 **	
	mDM			1.0 ± 0.6
Aspartate aminotransferase nmol/min/mg protein	Control/Sham		0.2 ± 0.05	0.4 ± 0.2
	50 mM glucose		0.2 ± 0.05	
	mDM			0.5 ± 0.2
Hexokinase µmol/min/mg protein	Control/Sham			26.0 ± 7.0
	mDM			59.0 ± 33.0
Citrate synthase µmol/min/mg protein	Control/Sham		34.3 ± 4.0	44.4 ± 19.6
	50 mM glucose		41.8 ± 5.3 **	
	mDM			35.4 ± 15.4
Aconitase µmol/min/mg protein	Control/Sham	36.4 ± 9.3	23.0 ± 7.2	31.5 ± 11.1
	0.2 mM SNAP	48.4 ± 12.7 *		
	50 mM glucose		30.0 ± 6.5	
	mDM			26.8 ± 16.9
Isocitrate dehydrogenase µmol/min/mg protein	Control/Sham	68.3 ± 22.3	56.7 ± 11.1	14.7 ± 7.1
	0.2 mM SNAP	80.2 ± 34.7		
	50 mM glucose		61.9 ± 8.4	
	mDM			9.6 ± 5.8

Data are means ± SD from 12–16 (for in vivo studies) or 5–14 (for in vitro studies) observations per group. Significantly different from the control: * (*p*–value < 0.05), ** (*p*–value < 0.01). Abbreviations: SNAP: *S*-Nitroso-*N*-acetylpenicillamine

**Table 2:** Metabolite levels measured in Wistar rat brains.

Parameter	Added	Primary Neurons	Neural Stem Cells	Wistar Rat’s Brain Tissue
Pyruvate nmol/mg protein	Control/Sham	50.7 ± 27.0	24.9 ± 8.5	13.2 ± 7.4
	0.2 mM SNAP	25.3 ± 15.5 **		
	50 mM glucose		75.2 ± 23.7 **	
	mDM			10.3 ± 5.5
Lactate nmol/mg protein	Control/Sham	15.3 ± 4.3	10.7 ± 5.5	25.0 ± 11.3
	0.2 mM SNAP	49.5 ± 15.2 ***		
	50 mM glucose		5.7 ± 5.1	
	mDM			35.3 ± 12.3 **
Citrate nmol/mg protein	Control/Sham	45.8 ± 13.	26.4 ± 9.3	
	0.2 mM SNAP	16.9 ± 11.2 **		
	50 mM glucose		10.4 ± 4.7 ***	
Oxaloacetate nmol/mg protein	Control/Sham	5.1 ± 0.6	2.3 ± 0.9	0.5 ± 0.4
	0.2 mM SNAP	3.1 ± 2.5 *		
	50 mM glucose		2.8 ± 1.5	
	mDM			0.4 ± 0.1

Data are means ± SD from 14–16 (for in vivo studies) or 5–12 (for in vitro studies) observations per group. Significantly different from control: * (*p*–value < 0.05), ** (*p*–value < 0.01), *** (*p*–value < 0.001).

**Table 3:** General characteristics of rats used in this project.

Parameters	Sham Control	Moderate DM	Severe DM
Body weight (g)	307 ± 33	210 ± 16 ***	240 ± 50 *
Blood glucose (mg/dL)	127 ± 13	513 ± 56 ***	523 ± 92 ***
Urine acetoacetate (µmol/24 h)	0.3 ± 0.1	1.9 ± 1.0 **	5.8 ± 2.4 ***

Significantly different from the sham control: * (*p*–value < 0.05), ** (*p*–value < 0.01) and *** (*p*–value < 0.001). Data are means ± SD from 6–17 animals per group.

**Table 4:** A list of primers and TaqMan probes used in this project.

Gene Transcript	Primers	TaqMan Probe	Transcript of Reference Gene
*Nat8l NM_001191681.1*	(F) tggctgacattgaacagtactaca (R) cacaacattgccgtccag	Universal ProbeLibrary Probe #83 (Roche, Cat #04689062001)	Universal ProbeLibrary Rat Actb Gene Assay
*Chat NM_001170593.1*	(F) gaagcttccaagccactttc (R) gtagtagagcctcagacgacgac	Universal ProbeLibrary Probe #66 (Roche, Cat #04688651001)	(Roche, Cat #05046203001)

**Table 5:** A list of antibodies used in this project.

Target Protein	Type of Antibody	Company	Cat#
β-actin	mouse primary monoclonal	Sigma Aldrich	A2228
NAT8L	rabbit primary polyclonal	Thermo Fisher Sc.	PA5-49536
Bcl-2	rabbit primary polyclonal	Abcam	Ab59348
β-III-tubulin	rabbit primary monoclonal	Cell Signaling	5568T
Caspase-3	rabbit primary polyclonal	Cell Signaling	9662s
ChAT	rabbit primary polyclonal	MyBioSource	MBS127981
CNPase	mouse primary monoclonal	Sigma Aldrich	C5922
GFAP	rabbit primary polyclonal	DAKO	Z0334
GAPDH	mouse primary monoclonal	Abcam	ab8245
Goat IgG	rabbit secondary polyclonal AP–conjugated	Sigma Aldrich	A4187
CHT-1	rabbit primary polyclonal	MyBioSource	MBS129733
Mouse IgG	goat secondary polyclonal AP–conjugated	Sigma Aldrich	A3562
Mouse IgG1	goat secondary polyclonal 488–conjugated	Thermo Fisher Sc.	A21121
NeuN	rabbit primary monoclonal	Cell Signaling	24307T
nAChR	rabbit primary polyclonal	Santa Cruz SCBT	sc-5591
PARP	rabbit primary polyclonal	Millipore	AB16661
Rabbit IgG	goat secondary polyclonal AP–conjugated	Santa Cruz SCBT	sc-2007
Rabbit IgG	goat secondary polyclonal 555–conjugated	Thermo Fisher Sc.	A21428
Synaptophysin	rabbit primary polyclonal	Abcam	ab14692
VAChT	goat primary polyclonal	Thermo Fisher Sc.	OSG00003W

## Supplementary Materials

Supplementary materials can be found at https://www.mdpi.com/1422-0067/21/22/8541/s1.

## Abbreviations

Aco	Aconitase
AspAT	Aspartate aminotransferase
b.w.	Body weight
Casp-3	Caspase-3
ChAT	Choline acetyltransferase protein
*Chat*	Choline acetyltransferase gene
CHT1	High affinity choline transporter
CNPase	2′,3′-cyclic-nucleotide 3′-phosphodiesterase
DIV	Days in vitro
DM	Diabetes mellitus
GFAP	Glial fibrillary acidic protein
IDH	Isocitrate dehydrogenase
LDH	Lactate dehydrogenase
mDM	Moderate diabetes mellitus
MTT	Methylthiazolyldiphenyl-tetrazolium bromide
NAA	*N*-acetylaspartate
NAT8L	Aspartate *N*-acetyltransferase protein
*Nat8l*	*N*-acetyltransferase 8 Like gene
NO	Nitric oxide
NSC	Neural stem cells
PDHC	Pyruvate dehydrogenase complex
SC	Citrate synthase
sDM	Severe diabetes mettilus
SNAP	*S*-Nitroso-*N*-acetylpenicillamine
STZ	Streptozotocin
TBARS	Thiobarbituric acid reactive substances
VAChT	Vasicular acetylcholine transporter
