# Supplementary material for: Neither Excessive Nitric Oxide Accumulation nor Acute Hyperglycemia Affects the N-Acetylaspartate Network in Wistar Rat Brain Cells
Source: Int J Mol Sci. 2020 Nov 12;21(22):8541. doi: 10.3390/ijms21228541 (PMC7697070; doi:10.3390/ijms21228541)
Supplement: Supplementary file 1 [file ijms-21-08541-s001.pdf]

Table 1S. A list of compounds used in this study.

NAME	COMPANY	Cat#
0.4% Trypan Blue solution	Sigma Aldrich	T8154
POPOP	Sigma Aldrich	P3754
1,4-Dioxan	Sigma Aldrich	292300119
2-Chloroacetamide	Sigma Aldrich	C0267
2-Thiobarbituric acid	Sigma Aldrich	T5500
PPO	Sigma Aldrich	D210404
4-Ethynylpyridine hydrochloride	Sigma Aldrich	530921
Acetic acid	POCH	568760114
Acetonitrile	POCH	10265456
Acetyl-CoA [acetyl-1-14-C] 50 µCi	Perkin Elmer	NEC313050UC
Adenosine	Sigma Aldrich	A4036
ADP sodium salt	Sigma Aldrich	A6646
Alcohol dehydrogenase	Sigma Aldrich	A7011
Albumin, bovine serum	Sigma Aldrich	A4503
AraC	Sigma Aldrich	C6645
Aspartic acid L-[U-14-C] 50 µCi	Perkin Elme	NEC268E050UC
ATP disodium salt	Sigma Aldrich	A2383
β-hydroxybutyrate dehydrogenase	Sigma Aldrich	H9408
B27 supplement	ThermoFisher Sc	17504044
B27 minus antioxidants supplement	ThermoFisher Sc	10889038
Basic fibroblast growth factor (bFGF)	ThermoFisher Sc	13256-029
BCIP	Sigma Aldrich	B6149
Brilliant blue G	Sigma Aldrich	27815
CHAPS	Sigma Aldrich	C9426
Choline chloride	Sigma Aldrich	C7527
Chloroform	POCH	BA4431116
cis-Aconitic acid	Sigma Aldrich	A3412
Citrate lyase	Sigma Aldrich	C0897
Citrate synthase	Sigma Aldrich	C3260

Coenzyme A sodium salt	Sigma Aldrich	C3144
D-Glucose	Sigma Aldrich	G5767
DAF-2 DA	Calbiochem	251505-M
Diethyl ether	Sigma Aldrich	309966
Diphenyloxazole	Sigma Aldrich	D210404
Dithiothreitol	Sigma Aldrich	D9779
DMEM/F12 + Glutamax	ThermoFisher Sc	31331-028
DMF	Sigma Aldrich	D4551
DTNB	Sigma Aldrich	D8130
EDTA	Sigma Aldrich	E1644
Epidermal growth factor (EGF)	VWR	BDAA354010
Eserine salicylate salt	Sigma Aldrich	45720
Ethanol	Sigma Aldrich	493511
Glucose-6-phosphate dehydrogenase	Sigma Aldrich	G8404
Glutamax	ThermoFisher Sc	3505006
Glutamic-Oxalacetic Transaminase	Sigma Aldrich	G2751
Glycerol	Sigma Aldrich	G2025
Glycine	Sigma Aldrich	G8898
Griess reagent	Sigma Aldrich	G4410
H_3PO_4	Sigma Aldrich	79617
HBSS	ThermoFisher Sc	4442135
HCl	POCH	575283721
$HClO_4$	Fluka	77228
HEPES	Sigma Aldrich	H4034
HEPES buffer	ThermoFisher Sc	15630-056
HEPES sodium salt	Sigma Aldrich	H3784
IgG standard	Sigma Aldrich	I4506
Isocitrate trisodium salt	Sigma Aldrich	I1252
Isocitrate dehydrogenase	Sigma Aldrich	I2002
α-Ketoglutarate disodium salt	Sigma Aldrich	75892
KCl	POCH	739740114
K_2HPO_4	Sigma Aldrich	P5504

KH_2PO_4	Sigma Aldrich	P5655
K_3PO_4	POCH	742020112
$KHCO_3$	Sigma Aldrich	237205
KOH	Sigma Aldrich	P5958
α-Ketoglutarate	Sigma Aldrich	75892
L-Aspartate	Sigma Aldrich	A6683
L-Glutamate	Sigma Aldrich	G5889
L-Glutamine	Sigma Aldrich	G8540
Lactic dehydrogenase	Sigma Aldrich	L-2500
Laemmle Sample Buffer	Bio-Rad	161-0737
Laminin	ThermoFisher Sc	23017015
Lithium potassium acetyl phosphate	Sigma Aldrich	0,1409
Maleic anhydride	Fluka	63200
Malic acid	Sigma Aldrich	M1000
Malate dehydrogenase	Sigma Aldrich	M2634
Methanol	Sigma Aldrich	621995156
$MgCl_2$	Sigma Aldrich	M8266
MOPS	Sigma Aldrich	M1254
MTT	Sigma Aldrich	M2128
N-acetylaspartic acid	Sigma Aldrich	A5625
NaCl	POCH	794121116
NAD	Sigma Aldrich	N3014
NADH	Sigma Aldrich	N8129
NADP	Sigma Aldrich	N5755
NADPH	Sigma Aldrich	N1630
NaOH	Sigma Aldrich	S8045
Nerve growth factor (NGF-β)	Sigma Aldrich	SRP4304
Neurobasal Media	ThermoFisher Sc	21103049
NH_4Cl	Sigma Aldrich	A9434
Nitrotetrazolium Blue chloride	Sigma Aldrich	N6876
PBS (sterile buffer)	ThermoFisher Sc	14190086
Penicillin-Streptomycin solution	Sigma Aldrich	P4333

Phenazine methosulfate (PES)	Sigma Aldrich	P9625
Phosphotransacetylase (PTA)	Sigma Aldrich	P2783
Poly-L-ornithine	Sigma Aldrich	P4957
Protease inhibitor cocktail	Sigma Aldrich	P8340
RNAlater®	Sigma Aldrich	R0901
S-Nitroso-*N*-acetylpenicillamine (SNAP)	TOCRIS	0598
SDS	Sigma Aldrich	L5750
Sodium oxalate	Sigma Aldrich	71800
Sodium phosphate	Sigma Aldrich	S0876
Sodium pyruvate	Sigma Aldrich	P2256
Spectra™ Multicolor Protein Ladder	ThermoFisher Sc	26623
Streptozotocin	Sigma Aldrich	S0130
Sucrose	Sigma Aldrich	S9378
Ultrapure distilled water	ThermoFisher Sc	10977035
Unstained Natural Protein Standards (Protein ladder, low range)	Bio-Rad	161-0304
TCA	Fluka	91228
Tetrabutyloammonium bisulfate	Sigma Aldrich	86853
Tetraphenylborate sodium	Sigma Aldrich	T25402
Thiamine hydrochloride	Sigma Aldrich	T4625
Thiobarbituric acid	Sigma Aldrich	T5500
Toluene	Sigma Aldrich	24529
TRIS Base	Sigma Aldrich	252859
Triton X-100	Sigma Aldrich	T8787
Tween 20	Sigma Aldrich	P9416

Supplement 1

Metabolic assays

To analyze metabolic profile in the cell lines, from each dish two independent supernatants were collected and reported as a one average result. To analyze brain tissue metabolic profile, 3 tissue supernatants per one brain were collected and then metabolic profile of each supernatant was measured in two independent samples. Eventually, 3 average results per 1 brain were reported in this study.

ATP, ADP, AMP and adenosine levels were assayed by RP-HPLC method [65]. Briefly, supernatant (pH = 7.0) was centrifuged at Micro Spin filter (Teflon membrane, Ø 0.22 mm, CIRO, Cat# CIPT-02). 25 μL of supernatant (50 μg of cell homogenate protein) was analyzed in a pre-column protected Hypersil^{TM} ODS C18RP column (150 x 4.6, i.d., MZ-Analysentechnik GmbH, Cat# 6045) by Flexar HPLC system (Perkin Elmer). Mobile phase A (10mM TBAHS / 100mM phosphate buffer, pH = 7.0) and mobile phase B (30% methanol) were mixed with flow rate: 1 mL/min under gradient program: 0 – 10 min (98% mobile phase A), 10 – 25 min (linear gradient form 98% to 0% mobile phase A), 25 – 40 min (0% mobile phase A), 40 – 45 (linear gradient form 0% to 98% mobile phase A). Separation time: t_{Ade} = 8.5 min, t_{ADP} = 20.0 min, t_{ATP} = 22.0 min [68].

Citrate level was determined using NADH/NAD conversion technique, at 340 nm and 37 ^{0}C. The reaction buffer contained 0.1 M Tris-HCl (pH=7.4), 0.1 mM NADH, 0.2 U MDH and neutralized supernatant (deproteinized from 40 μg of cell protein) in a final volume of 0.7 mL. The assay was initiated by the addition of 10 μL of 0.1 U citrate lyase (EC 4.1.3.6) [67].

MTT test was used to calculate proliferation as a total mitochondria activity. Cells were seeded at 48-well plates and cultured as usually. As soon as the cell culture was completed, 0.6 mL of fresh media with 2 mM Glutamax and 5 mg/mL MTT only (without supplement or other factors) was added to each well, followed by 3 h incubation in light – protected cell culture conditions. In order to dissolve the formed formazan, the cells were lysed by 0.3 mL of lysing buffer (50% DMF, 20% SDS, pH = 4.7, overnight). Finally, the formed formazan was monitored at 690 nm [41].

Nitric oxide cellular level was determined using diaminofluorescein-2 diacetate (DAF-2) membrane permeable fluorescent dye method with λ_{ex}=488 nm, λ_{em}=520 nm. Cells were seeded as usual, but we used fluorescence – dedicated black 96-well plates

instead of regular once. As soon as the cell culture was completed, 100 μL of fresh media with Glutamax and 2 μM DAF-2 AM only (without supplement or other factors) was added to each well followed by 1 h incubation at standard cell culture conditions. Next, the wells were washed 3 times with sterile PBS. Finally, to measure cellular nitric oxide linked with fluorescence dye, the cells were lysed for 15 min by lyses buffer containing 50 mM HEPES, 5 mM dithiothreitol, 0.1 mM EDTA and 0.1% CHAPS [69].

Nitric oxide in media level was determined used Griess modified method at λ= 540 nm and room temperature. 0.5 mL of media was mixed with 0.5 mL Griess reagent. After 15 min of room temperature incubation, absorbance was measured [70].

NAD and NADH levels were determined using MTT conversion technique, at λ= 570 nm (room temperature). Supernatants obtained with 0.2 M KOH were considered as having only NADH, while deproteinized with 0.1 HCl were consider as a total amount of NAD (NAD+NADH). Hence, NAD was calculated as result of subtraction of NADH from total NAD level. The reaction buffer contained 0.1 M Tris-HCl buffer (pH = 7.4), 0.04 M EDTA. 1 mM MTT, 1 mM PES, 5 mM ethanol and supernatant (100 μg of cell homogenate protein) in a final volume of 1 mL. Reaction was initiated by the addition of 10 μL of 40 U alcohol dehydrogenases [71].

Figure 5A

Cell line	Primary neurons (SNAP, 6 DIV)					NSC (glucose, 6 DIV)			
Concentration [mM]	0	0.1	0.2	0.4	0.8	25	37.5	50	75
	7				26	5	15	22	25
	10				16	11	18	26	22
	12				17	10	19	30	26
	13				20	7			
	6				16	6			
	9	11			34	6			
	11	11				6			
	12	18				9			
	11	9							
	10	10							
	11		11						
	12		9						
	19		12						
	13		7						
	12		12						
	11			10					
	12			10					
	19			18					
	27			11					
	14			11					

Figure 5B

Cell line	Primary neurons (SNAP, 3 DIV)					Primary neurons (SNAP, 6 DIV)					NSC (glucose, 6 DIV)			
Concentration [mM]	0	0.1	0.2	0.4	0.8	0	0.1	0.2	0.4	0.8	25	37.5	50	75
	100	91	18	22	11	100	71	50	40		100	82	85	100
	100	72	75	22	10	84	76	44	26		91	87	81	100
	100	68	70	30	12	100	88	39	29		100	86	83	97
	100		48		40	86	80	72	72					
	100		15			87	85	62						
	87													
	100													
	100													

Figure 5 C

Time [h]	1					3					6					24					72					144				
SNAP [mM]	0	0.1	0.2	0.4	0.8	0	0.1	0.2	0.4	0.8	0	0.1	0.2	0.4	0.8	0	0.1	0.2	0.4	0.8	0	0.1	0.2	0.4	0.8	0	0.1	0.2	0.4	0.8
	10	30	16	10	32	23	16	19	26	12	35	20	69	34	100	18	20	23	30	18	34	8	18	20	2	14	19	19	21	15
	7	10	3	9	2	38	31	13	63	41	39	18	18	25	100	25	7	32	50	46	35	7	33	2	21	12	30	29	27	26
	4	3	4	5	6	19	10	2	10	21	32	11	2	13	81	23	67	90	88	100	8	12	12	14	5	15	9	17	18	13
	28	9	40	17	51	28	33	29	36	23	44	49	4	22	218	22	66	77	100	91	21	22	17	19	15	20	24	22	20	23
	32	10	13	5	7	21	49	56	24	89	29	25	71	26	21	39	64	47	43	43	20	32	32	33	27	44	35	31	55	84
	40	2	4	23	12	29	40	49	39	52	19	24	21	22	100	49	28	74	43	83	49	26	13	26	5	25	23	28	34	27
	59	59	61	66	32	56	15	20	15	78	22	28	35	42	21	4	30	75	99	36	58	21	41	37	36	63	49	60	63	42
	65	79	67	4	45	39	12	10	98	39	43	38	31	46	26	45	60	48	64	90	60	23	27	53	2	60	65	69	67	51
	62	26	16	69	45	65	70	81	22	84	31	33	26	37	44	32	47	31	55	100	43	50	45	10	44	10	65	33	50	44
	16	1	7	19	6	10	16	19	17	15	29	15	5	18	100	6	48	58	100	50	12	23	12	2	2	16	15	17	17	14
	44	46	4	62	13	37	23	49	67	23	41	24	3	28	33	45	62	69	23	78	31	27	29	35	29	56	61	62	17	36
	64	4	17	78	40	28	16	30	18	15	31	34	41	11	52	32	31	41	82	34	42	47	44	19	43	75	8	75	67	48
	33	34	52	35	41	7	38	4	57	61	15	18	17	59	47	44	51	56	70	64	19	45	39	50	31	25	38	30	59	26
	34	27	42	43	39	39	52	55	42	64	41	53	11	100	67															
	37	44	53	53	45	40	54	56	56	55	21	64	70	100	62															
	50	34	35	31	37	33	36	49	34	46	38	16	51	100	66															
	37	46	47	58	55	51	56	48	78	68	47	64	39	100	93															
	44	48	40	72	67	45	67	35	100	79	9	32	8	39	64															

Figure 5 D

Time [h]	1					2					3				
SNAP [mM]	0	0.1	0.2	0.4	0.8	0	0.1	0.2	0.4	0.8	0	0.1	0.2	0.4	0.8
	3	0	21	137	152	0	80	140	158	165	0	37	100	189	297
	11	18	49	136	132	0	104	152	154	192	0	43	160	146	377
	9	4	9	161	155	0	145	196	164	196	0	21	169	219	201
	7	15	27	137	121	0	53	142	155	157	8	0	71	135	195
	16		30	129	110	0		123	139	139	9		117	243	194
